# DTS-Depth: Real-Time Single-Image Depth Estimation Using Depth-to-Space Image Construction

**DOI:** 10.3390/s22051914

**Published:** 2022-03-01

**Authors:** Hatem Ibrahem, Ahmed Salem, Hyun-Soo Kang

**Affiliations:** 1 Department of Information and Communication Engineering, School of Electrical and Computer Engineering, Chungbuk National University, Cheongju-si 28644, Korea; hatem@cbnu.ac.kr (H.I.); ahmeddiefy@cbnu.ac.kr (A.S.); 2 Electrical Engineering Department, Faculty of Engineering, Assiut University, Assiut 71515, Egypt

**Keywords:** depth estimation, real-time processing, convolutional neural networks

## Abstract

As most of the recent high-resolution depth-estimation algorithms are computationally so expensive that they cannot work in real time, the common solution is using a low-resolution input image to reduce the computational complexity. We propose a different approach, an efficient and real-time convolutional neural network-based depth-estimation algorithm using a single high-resolution image as the input. The proposed method efficiently constructs a high-resolution depth map using a small encoding architecture and eliminates the need for a decoder, which is typically used in the encoder–decoder architectures employed for depth estimation. The proposed algorithm adopts a modified MobileNetV2 architecture, which is a lightweight architecture, to estimate the depth information through the depth-to-space image construction, which is generally employed in image super-resolution. As a result, it realizes fast frame processing and can predict a high-accuracy depth in real time. We train and test our method on the challenging KITTI, Cityscapes, and NYUV2 depth datasets. The proposed method achieves low relative absolute error (0.028 for KITTI, 0.167 for CITYSCAPES, and 0.069 for NYUV2) while working at speed reaching 48 frames per second on a GPU and 20 frames per second on a CPU for high-resolution test images. We compare our method with the state-of-the-art methods on depth estimation, showing that our method outperforms those methods. However, the architecture is less complex and works in real time.

## 1. Introduction

In computer vision, depth estimation is one of the key tasks employed in numerous applications such as 3D scene construction and understanding, medical 3D imaging and scanning, background/foreground separation, depth perception in self-driving cars and robots, and 3D graphics. Depth is traditionally estimated using either a stereo camera or an IR depth camera; however, these systems involve expensive instruments and high-speed GPU processors for depth determination. Of late, the need for high-speed computer vision has increased due to the requirement for fast processing in embedded devices and smart phones, including self-driving cars and real-time 3D reconstruction. Such high-speed processing requires lightweight and memory-efficient computer-vision algorithms based on modern convolutional neural networks (CNNs). The latest research on depth estimation has demonstrated the effectiveness of using CNN-based algorithms for depth estimation with high accuracy; however, most recent studies [1,2,3,4,5,6,7,8,9,10,11,12,13,14,15,16] have not taken into consideration the processing speed and the optimization of such models to be employed in embedded systems or low-resource devices with limited memory and processing ability. In this study, we focus on monocular depth estimation (MDE), in particular, which involves depth prediction using a single RGB image, instead of stereo depth estimation (SDE). The latest depth-estimation CNN models generally depend on encoder–decoder architecture for compressing the input image to a deep latent vector and constructing the depth map from the deep features subsequently. Although this approach is efficient in estimating depth with high accuracy, it is usually so computationally expensive that it may not be applied in real time or in limited-hardware-capability devices. Therefore, we address the problem of expensive computation in depth estimation by adopting the concept of space image construction [17] of the depth map from numerous small-scale deep feature maps directly without the need for a decoder network. As eliminating the need for the decoder part that is generally used to construct the depth map from deep features, we realized high-speed processing, enabling the proposed network to work in real time on a GPU or even a CPU.

The contributions of this study are summarized as follows:

- We propose a high-speed CNN approach for single-image depth estimation using lightweight and fast architecture through which we eliminate the need for the decoder stage generally used in encoder–decoder depth-estimation architecture and replace it with fast depth-map construction from low-resolution feature maps.
- We efficiently extend the depth-to-space (DTS) module originally used for image super-resolution tasks to semantic depth-map construction through heatmap aggregation.
- We prove by experiments that the proposed method can run fast enough for real-time applications (20 fps) on CPUs which are lower in computational power than commonly used GPUs.

**Figure 1:**
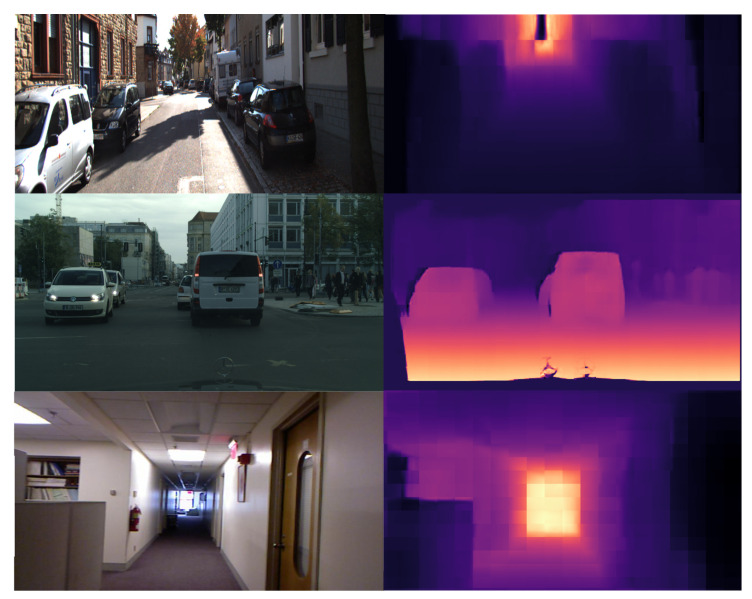
Example of the output depth predictions of the proposed method. Top-to-bottom, KITTI, Cityscapes, and NYUV2 RGB images and their corresponding predicted depth maps.

Figure 1 shows sample depth-estimation results obtained from the proposed method in different cases of outdoor and indoor scenes.

The structure of this paper is as follows. Section 2 summarizes the recent related work, Section 3 presents the implementation of the suggested method, Section 4 presents the datasets employed in our experiments, Section 5 shows the details of the experiments done, the obtained results from them, and the comparisons with other methods of depth estimation. Section 6 discusses the limitations of the proposed methods and the possible future work based on this research, and Section 7 gives the conclusions of this paper.

## 2. Related Work

Depth estimation is one of the earliest research areas in computer vision. One of the first methods to estimate depth was proposed by Torralba and Oliva [18], who demonstrated the relationship between the image spectral magnitude and depth variation. Chun et al. [19] proposed depth estimation in indoor scenes using ground plane information. Saxena et al. [20] proposed a depth-estimation method using the textural features of 3D objects in the scene using Markov random fields (MRF). An early and efficient CNN-based method was proposed by Eigen et al. [1], which involved a deep neural network model composed of two stages. The first stage predicted a coarse depth map using CNN architecture, whereas the second stage refined the map for fine depth estimation. Although the depth prediction was blurry, it was a significant beginning for monocular depth estimation. Based on their results, depth refinement techniques [2,3] were proposed to overcome the blurry depth in [1], using conditional random fields (CRF) based on an encoder–decoder architecture in which they employed superpixel segmentation to refine the depth prediction. Gan et al. [4] employed the affinity layer in a CNN encoder–decoder architecture to learn the global and local context of the image efficiently. Xu et al. [5] proposed a multiple-scale CNN based on cascaded CRF stage architecture to fuse the best features. Other studies [6,7,8] employed stereo reconstruction loss to obtain the monocular depth using the disparity consistency based on unsupervised and semi-supervised methods. Godard et al. [6] employed a stereo-pair image to estimate the depth using the consistency loss. Garg et al. [7] applied a simple warping technique to predict the disparity map using a deep CNN. Kuznietsov et al. [8] proposed the image alignment loss to improve the depth-map quality. Cao et al. [9] proposed depth estimation applying pixel-wise classification loss to obtain the pixel-wise confidence depth values in a probability distribution form. Subsequently, Cao et al. [10] proposed a technique to pre-train a deep CNN on the relative depth obtained by stereo matching the images, and further fine-tuned the network to estimate the monocular depth. Fu et al. [11] proposed an architecture based on atrous spatial pyramid pooling (ASPP) to extract the dense features and applied them to estimate accurate depth boundaries through regression. Zuo et al. [12] proposed a depth enhancement technique based on multiscale guidance to local and global residual learning based on pixel intensity. Mohaghegh et al. [13] presented a mapping technique from the image patches to the depth predictions by refining a pre-trained model, which learned the global form of the depth maps. Ma et al. [14] proposed direct 3D reconstruction of the scene from a 2D image using an attention module based on separated channel-spatial convolution, which extracted the 3D representation of objects through an adaptive channel and spatial fusing. Bhat et al. [15] presented a transformer-based encoder–decoder architecture called Adabins, which learned the depth by dividing the depth range into bins whose center was estimated adaptively per image. Ranftl et al. [16] suggested the application of vision transformers as the backbone for dense predictions, where tokens from the different stages of the vision transformer were assembled in an image-like representation using a convolutional decoder. Liu et al. [21] proposed a similar approach to ours in which they adopted fully convolutional multiscale dense network based on DenseNet169 [22] for monocular depth estimation. They also proposed a dense upsampling block that includes a sequence of convolutional filters followed by a pixel shuffle operation to obtain higher resolution output. The pixel shuffle operation works in a similar way to DTS but with a different technique since they apply four 3×3 convolutional filters before the pixel shuffle. Their method showed a poor performance in terms of delta accuracy ($\delta_{1}$ = 0.836) and speed (the model’s number of parameters is 52 million parameters so that model cannot realize real-time processing). Zioulis et al. [23] proposed a CNN with an encoder and a hierarchical decoder with four different branches that construct the depth at different scales using a combination of predicted coarse and fine depth features.

Although CNN-based encoder–decoders have successfully performed high-accuracy depth estimation, some drawbacks in depth construction using the decoder remain. In most cases, the constructed depth is coarse and has blurry boundaries, and always needs extra refining stages to realize accurate depth prediction. These extra stages add more complexity and cause high latency in the inference step. We propose to directly estimate the depth from the encoder stage because we can construct a depth map of the same size as the input image from the small-scale features, using DTS image construction. For performance evaluation, we compare our method with the state-of-the-art methods on depth estimation and furthermore, we will consider FastDepth [24] which is an encoder–decoder depth-estimation method based on MobileNet, since it works accurately and efficiently on embedded devices at high speed. Although our method is slightly slower than FastDepth, it outperforms FastDepth in terms of the RMSE error and delta accuracy.

## 3. Proposed Method

The proposed method aims to construct the depth map directly using an encoder architecture employing DTS image construction, as detailed in the next subsection. The architecture used is a modified MobileNetV2 architecture, which is small and lightweight with fewer parameters and multiplication/addition computations (MACs).

### 3.1. DTS Image Construction Implementation Details

**Figure 2:**
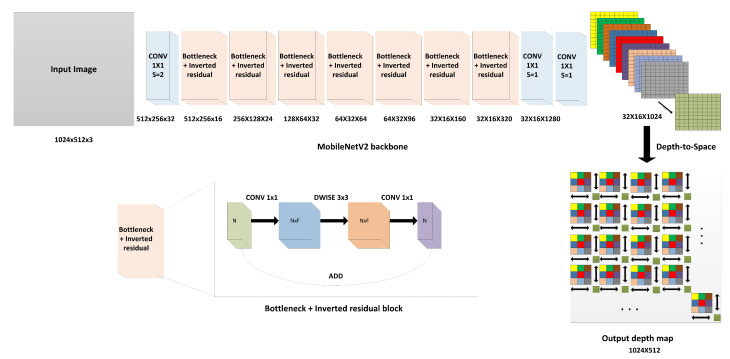
The architecture of the proposed method. With the modified MobileNetV2 architecture, the DTS layer (module) is added after the last convolutional layer to arrange the pixels in the 32 × 16 × 1024 low-resolution heatmaps as a (32 × 32) × (16 × 32) superpixel, which equals 1024 × 512. The bottleneck and inverted block consists of an expansion module which expanded the depth of input features by a factor F (an integer number), followed by depthwise separable convolution, and finally, compression is applied to the features by the projection module with the same factor F. CONV and DWISE refer to convolutional and depthwise separable convolutional layers, respectively.

The DTS module (or sub-pixel convolution layer) was first proposed by Shi et al. [17] in their efficient sub-pixel CNN, which was devised to perform super-resolution for single images and videos in real time. This method showed highly accurate results in super-resolution. It could eliminate high complexity in the previous architectures traditionally used for image super-resolution by reducing the architecture to only three convolutional layers with a gradual increase in the layer depth, followed by the construction of the high-resolution output image through low-resolution feature aggregation. In our case, we construct a depth map instead of an image. Low-resolution feature aggregation is performed by rearranging the elements of tensor *H* × *W* × $r^{2}$ to a tensor of shape rH × rW, where *H*, *W*, and *r* are the feature map height, width, and depth, respectively. This operation can be expressed mathematically as in Equation (1):

(1) $$D_{x,y}=T_{\left[x/r],[y/r],r.mod(y,r)+mod(x,r\right)},$$

where $D_{x,y}$ is the constructed depth map, *T* is the feature map at the layer before the DTS layer, *r* is the feature map depth, and operation mod is the modulus. Equation (1) maps the pixel from the low-resolution feature maps to the depth maps when the condition mod(x,r)=0 or mod(y,r)=0 is true through a learnable process. The DTS module is depicted in Figure 2 in detail. The construction loss function used to learn the final depth map is the mean absolute error function, as shown below:

(2) $$Loss_{x,y}=\frac{1}{r^{2}HW}\sum_{x=1}^{rW}\sum_{y=1}^{rH}{\left(D_{x,y}^{GT}-D_{x,y}^{Constructed}\right)}^{2},$$

where $D_{x,y}^{GT}$ is the ground-truth depth map and $D_{x,y}^{Constructed}$ is the constructed depth map.

We used the above-mentioned DTS module to construct the final depth map from numerous small-scale encoded feature maps, each of which contains detailed features at a slightly different position. In addition, the encoding architecture was selected to be small-sized and lightweight for realizing high processing speed.

### 3.2. Modified MobileNetV2 Architecture

MobileNetV2 proposed by Sandler et al. [25] of Google Inc. is a CNN, which has been well optimized to work on limited-capability devices such as mobile phones. The architecture is composed of 53 layers employing the so-called linear bottlenecks and inverted residuals. The linear bottlenecks include an expansion module (1 × 1 convolution with more output filters), followed by depthwise separable convolution (which is depthwise spatial convolution acting on each channel separately), and finally, a projection module (1 × 1 convolution with lesser output filters). Expansion and projection are performed with a factor, which is an integer multiple of the feature map input channels; the inverted residual layer is a low-dimensional subspace encoding layer, which enables memory-efficient implementation. Figure 2 shows the block diagram of the bottleneck and inverted residual module. MobileNetv2 has fewer parameters (3.4 million parameters) and FLOP count (0.3 Giga FLOPs), which realize a high accuracy of 0.901 as the Top 5 accuracy on the challenging ImageNet [26] classification dataset.

**Figure 3:**
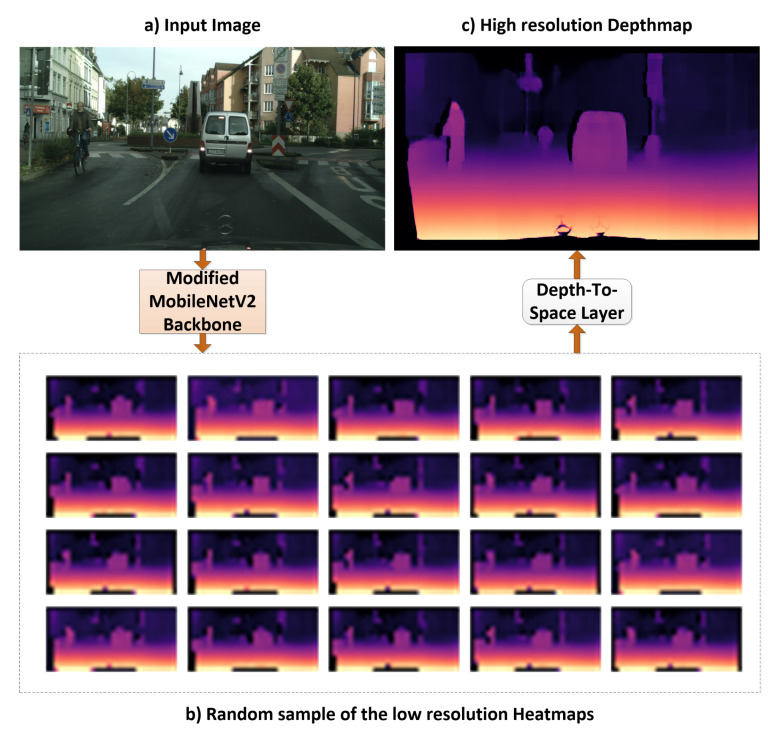
Visualization of the low-resolution heatmaps learned by the modified MobileNetV2 architecture from a Cityscapes test image: (**a**) Input image of size 1024 × 512, (**b**) randomly selected low-resolution heatmaps after the last 1 × 1 × 1024 convolutional layer of size 32 × 16 and (**c**) high-resolution depth map after the low-resolution heatmaps are aggregated in the DTS layer.

We modify the MobileNetV2 architecture by removing the last two layers, which are the fully connected layer and the global average pooling layer, and then we add a 1 × 1 convolution layer with 1024 filters because the image is spatially downscaled by a factor of 32. To obtain a depth map of the same size as the input image, the DTS module is added at the end to aggregate the pixels of the 1024 filters for constructing the final depth map sized $32^{2}$. This process is highly efficient with respect to speed and accuracy as it can rapidly perform pixel arrangement from the heatmaps to the final depth map with high accuracy. The output of the arrangement process is learnable as it allows a gradient flowing in the backpropagation during network training. Equation 3 shows the relationship between the constructed depth maps and the low-resolution heatmaps in the final layer before the DTS layer:

(3) $$D^{Constructed}=W_{L}*f^{L-1}\left(H^{LR}\right)+b_{L},$$

where $W_{L}$ and $b_{L}$ are the weights and biases in the DTS layer, $H^{LR}$ are the low-resolution heatmaps, and *f* is the activation function for the layer. Figure 3 displays 20 low-resolution heatmap samples (32×16 in the Cityscapes dataset with an input image sized 1024 × 512) obtained from MobileNetV2 after the 1 × 1 × 1024 convolutional layer and the reconstructed high-resolution depth map after the DTS layer. The low-resolution heatmaps are small depth maps with different depth details of the image, and the DTS layer aggregates the depth values from the low-resolution maps to form the high-resolution depth map depending on the learned weights and biases.

Among the lightweight architectures such as MobileNetV1 [27], ShuffleNet1.5 [28], and NasNet-A [29], MobileNetV2 was selected as the main architecture because its performance is optimal for our application considering the tradeoff between the few parameters/computations count and the high accuracy, as indicated in [25]. MobileNetV2 outperforms MobileNetV1 [27] in terms of the Top1 classification accuracy on ImageNet, with fewer parameters and multiplication/addition operations due to the use of linear bottlenecks and inverted residuals, whereas MobileNetV1 employs depthwise separable convolution and 1 × 1 convolution for projection with RELU6 activation. Moreover, it outperforms ShuffleNet1.5 [28] (which has approximately the same complexity as MobileNetv2) in terms of the Top 1 accuracy; both have the same number of parameters and multiplication/addition operations approximately. NasNet-A [29] is a NasNet version architecture with similar performance to MobileNet, ShuffleNet1.5, and MobileNetv2, and employs a stream of normal (a convolutional network that returns feature maps of the same input dimension) and reduction cells (a convolutional network that returns feature maps half the input size). Although NasNet-A [29] outperforms MobileNetV2 in terms of the Top 1 accuracy by 2%, it is 1.5 times and 1.9 times the number of parameters and multiplications/additions, respectively, as MobileNetV2; hence, the frame-processing speed of MobileNetv2 is 2.44 times faster than NasNet-A.

Table 1 compares MobileNet, ShuffleNet1.5, NasNet-A, and MobileNetV2 in terms of the Top 1 classification accuracy on the ImageNet dataset, the number of parameters, multiplication/addition count, and CPU processing time. The device used for testing is a Google Pixel 1 phone and the framework is TF-Lite as mentioned in [25]. According to [25], MobileNetV2 also attained higher mean average precision (mAP) for the object detection task than SSDLite (SSD [30] + MobileNetV1) and Yolov2 [31], with considerably fewer parameters, so that the computations are faster. In addition, MobileNetV2 outperforms MobileNet and ResNet-101 [32] as feature extractors for Deeplab [33] in semantic segmentation with respect to the mean intersection over union (mIOU), the number of parameters, and multiplication/addition count.

## 4. Datasets for Experiments

We trained and tested our proposed method on three different datasets, KITTI, Cityscapes, and NYUV2, which include depth data for both outdoor and indoor scenes.

### 4.1. Kitti Dataset

The KITTI dataset [34] is a large annotated dataset for several self-driving vehicle-related tasks such as object detection, semantic and instance segmentation, stereo depth estimation, monocular depth estimation, and 3D object detection. The dataset for depth data is calculated from point clouds acquired by a LIDAR sensor and is highly sparse, covering only 5% of the depth map. Hence, researchers generally interpolate the depth map with background interpolation techniques to fill in the depth map for training, whereas in the evaluation stage, they generally use the original sparse depth maps. We applied bilateral and median filtering for preprocessing the depth map, in addition to background interpolation to obtain dense depth maps. The dataset contains 23,297 images and their corresponding depth maps. We applied Eigen split [1] for splitting the dataset into training, validation, and test sets. Furthermore, we resized depth maps of 1224 × 375 to 608 × 224, in which the invalid depth pixels were cropped to accelerate the training process.

### 4.2. Cityscapes Dataset

The Cityscapes dataset [35] provides data that assists in the semantic understanding of urban street scenes, and annotations for several computer-vision tasks such as semantic and instance segmentation, depth estimation, and 3D vehicle detection. The depth is provided indirectly in the form of a disparity map calculated using a stereo camera. We train the network directly on the disparity map and then use the form provided along with the dataset to calculate the depth linearly from the disparity map, as follows:

(4) $$Depth_{x,y}=\frac{f_{x}*baseline}{Disp_{x,y}},$$

where $f_{x}$ and baseline are the focal length in the x-axis and the baseline for the stereo camera used for capturing the scene, respectively. $Disp_{x,y}$ is the disparity value of a given pixel.

The dataset contains 5000 training, validation, and test images as well as 20,000 extra training stereo-pair images (normal RGB scenes with the corresponding disparity). We trained our network on the left image of the stereo pair provided in the dataset, as we train the network for depth estimation using only a single input image. We used 24,500 images for training and 500 images for validation. The original image and disparity maps of 2048 × 1024 were resized to 1024 × 512 to accelerate the training process while maintaining high-resolution in the depth-estimation task.

### 4.3. Nyuv2 Dataset

**Figure 4:**
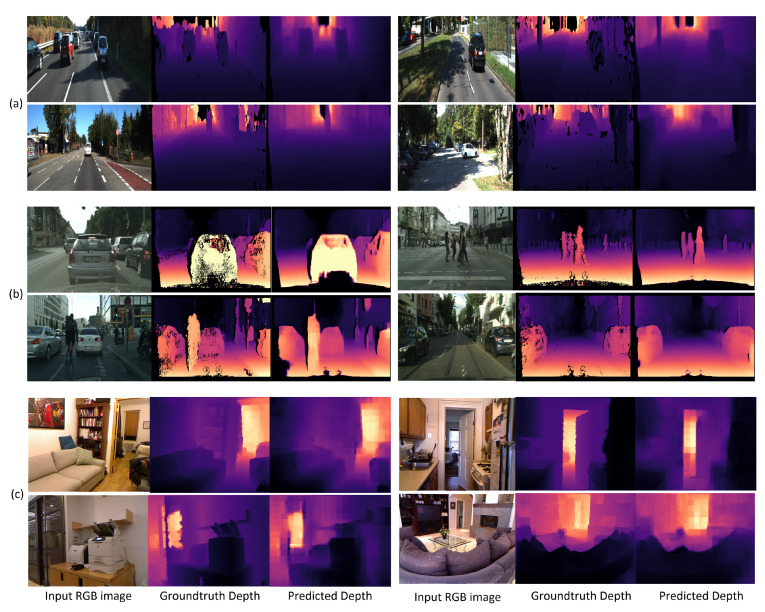
Sample results obtained through the proposed method: (**a**) results from KITTI test samples, (**b**) results from Cityscapes test samples, and (**c**) results from NYUV2 test samples. Each row has two samples of test results showing the input RGB image, ground-truth depth, and the predicted depth by the proposed method.

NYUV2 [36] is an indoor scene depth and segmentation dataset provided by a research group at New York University. It provides numerous indoor scenes collected from different indoor locations such as bedrooms, kitchens, basements, and bathrooms. The dataset was acquired by a Kinect sensor, which consists of RGBD images (RGB images and the depth map). The dataset contains 1449 labeled images and 407,024 raw images with a resolution of 640 × 480 for the images and depth maps. We trained our network on the clean labeled data only because the raw data includes many invalid depth pixels and noise from the shadows, and specular or low albedo surfaces in the scene. The dataset is split into 795 training and 654 test images. We trained our network without resizing the RGB images and depth maps to obtain high-resolution depth estimation. Figure 4 depicts sample depth-estimation results obtained using the proposed method on the KITTI, Cityscapes, and NYUV2 depth datasets.

## 5. Experimental Results

The proposed method was trained on three datasets, KITTI, Cityscapes, and NYUV2. The three trained models were evaluated on the test sets of the three datasets.

### 5.1. Training and Test Configurations

For training, we used a desktop PC with an Intel Core i7-8700 CPU at 3.2 GHz, NVIDIA RTX 3090 GPU, and 64-GB RAM. The training and testing image sizes are similar depending on the dataset as mentioned in the previous sections (608 × 224 for KITTI, 1024 × 512 for Cityscapes, and 640 × 480 for NYUV2). We tested our approach on different Nvidia GPUs (GTX1060, Titan xp, RTX2080, Titan RTX, RTX3090) and Intel CPUs (i7-7700, i7-8700, i7-9700, and i7-10700) to explore the speed capability of our approach. Tensorflow Keras was used to implement the CNN network, and the training of each model on the three different datasets was performed for 500∼1000 epochs with Adam’s optimizer. In all the training cases, the modified MobileNetv2 model was initialized with ImageNet [26] weights as we believe it speeds up the training process because of the prior classification features knowledge.

### 5.2. Evaluation Metrics

The metrics used for evaluating the depth estimation are as follows: the average absolute relative error (REL), squared relative difference (Sq_REL), root mean squared error (RMSE), and threshold accuracy $\delta_{i}$ of $y_{p}$, the mathematical expression of each metric can be stated such as in Equations (5)–(8):

(5) $$REL=\frac{1}{n}\sum_{p}^{n}\frac{|y_{p}-\hat{y_{p}}|}{y}$$

(6) $$Sq\_REL=\frac{1}{n}\sum_{p}^{n}\frac{||y-\hat{y}{\left|\right|}^{2}}{y}$$

(7) $$RMSE=\sqrt{\frac{1}{n}\sum_{p}^{n}{\left(y-\hat{y}\right)}^{2}}$$

(8) $$Delta\_accuracy\left(\delta_{i}\right)=max\left(\frac{y}{\hat{y}},\frac{\hat{y}}{y}\right)=\delta <thr$$

where *y* and $\hat{y}$ are the ground truth and predicted pixel values, *n* is the number of pixels in the depth map, and thr is a threshold value commonly set to three specific values ($thr=1.25,1.25^{2},1.25^{3}$).

### 5.3. Accuracy and Speed

Our model shows very low REL errors of 0.028, 0.167 and 0.069 on KITTI, Cityscapes, and NYUV2, respectively, (as reported in Table 2) while estimating a high-resolution depth map. Figure 4 displays sample results of the proposed method on the KITTI, Cityscapes, and NYUV2 depth datasets. The proposed method could realize a low frame-processing time on GPU (NVIDIA GTX1060, Titan XP, RTX 2080, Titan RTX, and RTX 3090) as well as CPU (Intel i7-7700, i7-8700, i7-9700, and i7-10700). Table 3 depicts the measured values of the different evaluation metrics and processing time on the different GPUs and CPUs. The proposed method realizes high speed in frame processing when using high-resolution images due to the well-optimized small architecture. NVIDIA RTX 3090 and Intel i7-10700 show the lowest processing time as expected, but still, the other GPUs and CPUs also show an excellent processing time even at high-resolution images. The processing time is expected to be considerably lower than the values shown in Table 3 if smaller images are used; however, we target high-resolution depth estimation instead of low-resolution depth, which contrasts with the latest studies that resize input images to smaller ones.

### 5.4. Comparison with the State-of-the-Art Methods

**Figure 5:**
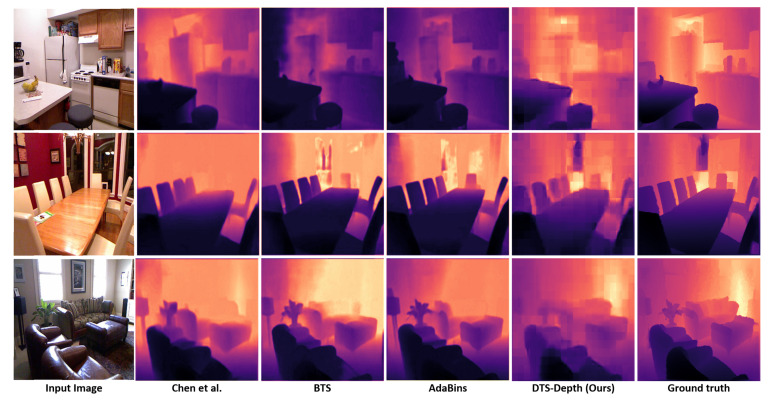
Quality comparison between the predicted depth by the proposed method (DTS-Depth), Chen et al. [43], BTS [45] and AdaBins [15], respectively on samples from NYUV2 test dataset. Our method has a high-quality depth estimation as it can predict the most similar depth map to the ground-truth depth map while other SOTA methods have some divergence from the ground truth depth.

We compared our results with the state-of-the-art methods with respect to the different error metrics and δ accuracy at different thresholds. The results showed that the proposed method produces lower depth error values than the other methods. As our model is trained on relatively high-resolution depth maps, it outperforms all the state-of-the-art methods on the three datasets in terms of delta accuracy. Table 2 demonstrates the comparison between the errors and accuracies obtained from the proposed method and those of several other recent methods on KITTI, Cityscapes, and NYUV2. Figure 5 shows a quality comparison between the predicted depth map by our method (DTS-Depth), Chen et al. [43], BTS [45], and AdaBins [15]. It is obvious that our method has the most similar depth map to that of the ground truth. Although there is a little blocking effect, which will be addressed in our future work, it does not have much effect on the accuracy.

### 5.5. Comparison with FastDepth

**Figure 6:**
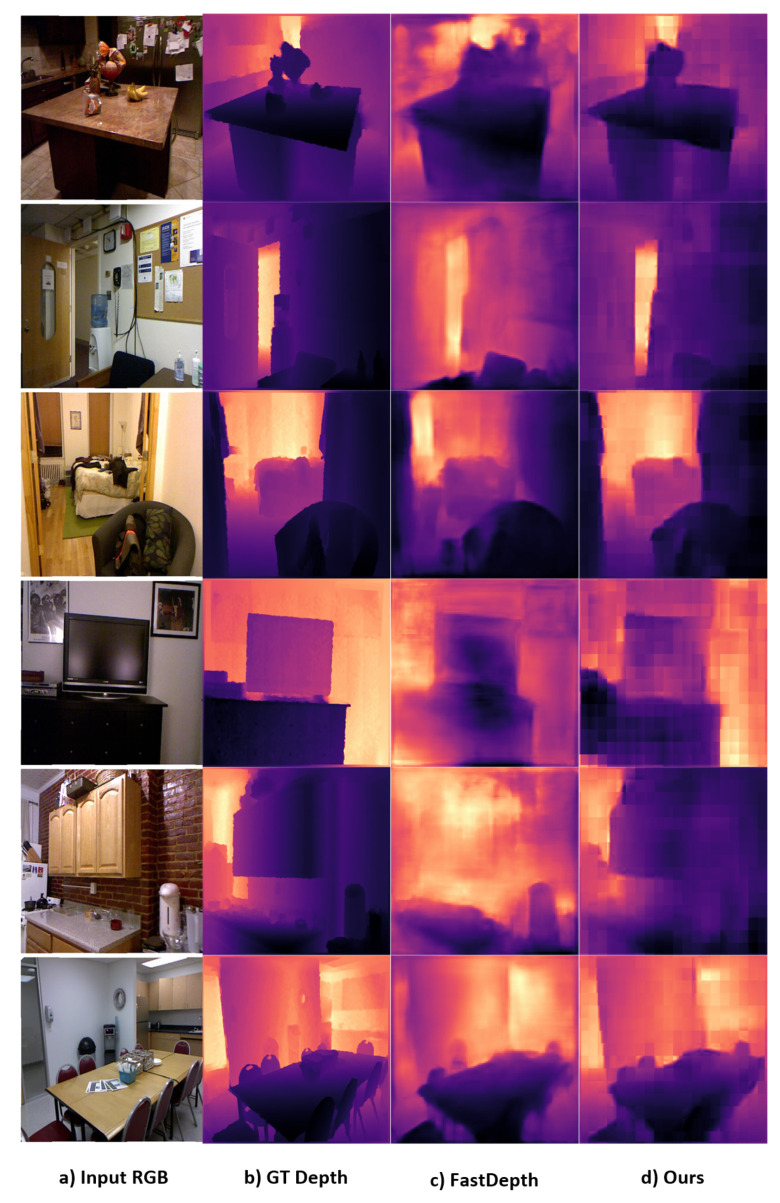
Quality comparison between our proposed method and FastDepth. Our method can predict the depth more clearly and accurately because we train using high-resolution depth maps. (**a**–**d**) are the input RGB, ground-truth depth map, FastDepth predicted depth map, and our predicted depth map, respectively.

We compare our method with FastDepth [24], in particular, because it is a fast depth-estimation method based on MobileNet. It employs a lightweight encoder–decoder architecture, which is appropriate for embedded devices. The model showed competitive accuracy and low errors because a low image resolution of 224×224 was used. In addition, The model showed competitive accuracy and low errors at a low image resolution of 224×224, i.e., the model was evaluated using a resized low-resolution ground truth of 224×224. In contrast, we trained and evaluated our method at an image size of 640 × 480. In general, evaluation at low-resolution results in lower error values and higher accuracy because the evaluation metrics is depending on the pixel count. Table 4 shows the comparison to FastDepth. Although the comparison is unfair because we use high-resolution images (640×480), the proposed method outperforms FastDepth in terms of the RMSE and $\delta_{1}$ accuracy, whereas our method consumes double the GPU and CPU processing time as shown in Table 4. Our model can achieve the same accuracy as that of a highly optimized model for embedded systems without requiring a decoder, using space-to-depth construction instead. FastDepth was evaluated on the NVIDIA Jetson board and CPU, and they proposed a MobileNetV1 encoder with a decoder configuration of depthwise + skip connections + feature additions. We compare the proposed method against the FastDepth architecture with a decoder configuration of depthwise + skip connections + feature additions. For a fair comparison with FastDepth, we performed it on one of the GPUs and CPUs used (NVIDIA RTX3090 and Intel Core i7-8700 CPU @ 3.2 GHz) for comparison to our method. Figure 6 demonstrates the quality of the predicted depth by our method and FastDepth.

## 6. Limitations and Future Work

Although the obtained results are good in terms of speed and accuracy, there is a limitation of the method represented by the blocking effect produced in the predicted depth, especially in the case of NYUV2 images. We believe that this drawback happens due to the single-stage upsampling process to the features produced by the encoder network; this process up-samples the features five times their size to achieve a depth map with the same size of the input image. Performing such large upsampling in a single stage produces this kind of artifact or blocking effect. However, the predicted depth values of the pixels are true and close to the ground-truth values. This problem can be solved in the future using multiple-upsampling decoder stages which will increase the complexity of the model and will definitely reduce the frame-processing speed, so the current model in this paper is still robust in terms of accuracy and speed regardless of the blocking effect, which is a minor problem. The architecture of our proposed method can be more improved to obtain higher accuracy and lower error values sacrificing the speed and employing deeper architectures such as ResNet [32], Xception [47], or EfficientNetB7 [48]. We focused in this research on keeping the encoder stage light by extracting the fewest possible features to realize real-time processing. In addition, the proposed model can be extended to perform semantic segmentation and instance segmentation because these tasks are similar to depth estimation in predicting image-like dense masks.

## 7. Conclusions

The proposed method showed that the DTS module originally proposed for the image super-resolution task could be efficiently extended for depth estimation with high accuracy, which is proved by the experimental results obtained. Moreover, it demonstrated that this concept could work well for high-resolution depth estimation, which is the outstanding merit of our work, considering that conventional depth-estimation methods are generally performed on low-resolution images. Our proposed method also solves the major problem of the high complexity of depth-estimation methods represented at the CNN encoder–decoder, as our method eliminates the need for the decoder stage and replaces it with the DTS module. Although the proposed method showed that it can work efficiently on Nvidia GPUs and Intel CPUs, it can work as well on devices with limited-capability processors because the architecture is extremely lightweight. Conclusively, our method is a good solution for fast depth estimation in applications such as self-driving vehicles, robots, and 3D medical imaging.

## Figures and Tables

**Table 1:** Comparison of MobileNetV1 [27], ShuffleNet1.5 [28], NasNet-A [29], and MobileNetV2 [25] in terms of the Top 1 classification accuracy on ImageNet, multiplication/addition computations (MACs), and CPU processing time on Google Pixel 1.

Network	Top1 acc. %	Params	MACs	CPU Time (ms)
MobileNetV1	70.6	4.2 M	575 M	113 ms
ShuffleNet1.5	71.5	3.4 M	292 M	-
NasNet-A	74.0	5.3 M	564 M	183 ms
MobileNetV2	72.0	3.4 M	300 M	75 ms

**Table 2:** Comparison of the performances on the KITTI, Cityscapes, and NYUV2 depth datasets. The original study results are reported.

Method	Dataset	REL	Sq Rel	RMSE	$\delta_{1}$	$\delta_{2}$	$\delta_{3}$
Eigen et al. [1]	KITTI	0.280	0.3012	8.734	0.702	0.898	0.967
	NYUV2	0.158	-	0.641	0.769	0.950	0.988
Laina et al. [37,38]	Cityscapes	0.257	4.238	7.273	0.765	0.893	0.940
	NYUV2	0.127	-	0.573	0.811	0.953	0.988
Xu et al. [5,38]	Cityscapes	0.246	4.060	7.117	0.786	0.905	0.945
	NYUV2	0.121	-	0.586	0.811	0.954	0.987
Liu et al. [21]	KITTI	0.127	-	4.977	0.838	0.948	0.980
	NYUV2	0.127	-	0.506	0.836	0.966	0.991
Hao et al. [39]	NYUV2	0.127	-	0.555	0.841	0.966	0.991
Lee et al. [40]	NYUV2	0.131	-	0.538	0.837	0.971	0.994
Fu et al. [11]	KITTI	0.072	0.307	2.727	0.932	0.984	0.994
	NYUV2	0.115	-	0.509	0.828	0.965	0.992
SharpNet [41]	NYUV2	0.139	-	0.502	0.836	0.966	0.993
Hu et al. [42]	NYUV2	0.115	-	0.530	0.866	0.975	0.993
Chen et al. [43]	NYUV2	0.111	-	0.514	0.878	0.977	0.994
Yin et al. [44]	KITTI	0.072	-	3.258	0.938	0.990	0.998
	NYUV2	0.108	-	0.416	0.875	0.976	0.994
BTS [45]	KITTI	0.059	0.245	2.756	0.956	0.993	0.998
	NYUV2	0.110	-	0.392	0.885	0.978	0.994
DPT-Hybrid [16]	KITTI	0.062	-	2.573	0.959	0.995	**0.999**
	NYUV2	0.110	-	0.357	0.904	0.988	**0.998**
Zhang et al. [38,46]	Cityscapes	0.234	3.776	7.104	0.776	0.903	0.949
	NYUV2	0.144	-	0.501	0.815	0.962	0.992
SDC-Depth [38]	Cityscapes	0.227	3.800	**6.917**	0.801	0.913	0.950
	NYUV2	0.128	-	0.497	0.845	0.966	0.990
AdaBins [15]	KITTI	0.058	0.190	2.360	0.964	**0.995**	**0.999**
	NYUV2	0.103	-	0.364	0.903	0.984	0.997
**DTS-Depth(Ours)**	KITTI	**0.028**	**0.152**	**2.256**	**0.967**	0.991	0.997
	Cityscapes	**0.167**	**1.639**	7.785	**0.804**	**0.921**	**0.958**
	NYUV2	**0.069**	**0.245**	**0.295**	**0.959**	**0.994**	**0.998**

**Table 3:** Evaluation of the speed of the proposed method on the KITTI (at an image size of 608 × 224), Cityscapes (CS at an image size of 1024 × 512), and NYUV2 (at an image size of 640 × 480) depth datasets using different NVIDIA GPUs and Intel CPUs. The clock speed, number of cores, number of threads, and frame-processing time for each GPU or CPU platform are indicated.

Platform	Clock Speed	#Cores	#Threads	KITTI Time	CS Time	NYUV2 Time
NVIDIA GTX1060	1506 MHz	1280	-	53 ms	103 ms	58 ms
NVIDIA Titan XP	1405 MHz	3840	-	50 ms	85 ms	51 ms
NVIDIA RTX 2080	1515 MHz	3072	-	35 ms	74 ms	38 ms
NVIDIA Titan RTX	1350 MHz	4608	-	31 ms	68 ms	35 ms
NVIDIA RTX 3090	1395 MHz	10,496	-	21 ms	68 ms	31 ms
Intel i7-7700	3.6 GHz	4	8	102 ms	231 ms	128 ms
Intel i7-8700	3.2 GHz	6	12	73 ms	173 ms	101 ms
Intel i7-9700	3.0 GHz	8	8	75 ms	201 ms	110 ms
Intel i7-10700	3.8 GHz	8	16	52 ms	155 ms	65 ms

**Table 4:** Comparison between the proposed method and FastDepth with respect to the RMSE, $\delta_{1}$, NVIDIA RTX3090 GPU time in milli-seconds (ms), and Intel i7-8700 CPU time in milli-seconds (ms).

Method (Image Size)	RMSE	$\delta_{1}$	GPU Time (ms)	CPU Time (ms)
FastDepth (224×224)	0.604	0.811	17	50
Ours (640×480)	0.295	0.959	31	101

## Data Availability

The datasets used in this paper are public datasets. We also provide the test and the evaluation codes of the proposed method at: https://github.com/HatemHosam/DTS-Depth (accessed on 15 November 2021).

## References

[1] Eigen D., Puhrsch C., Fergus R. (2014). Depth map prediction from a single image using a multi-scale deep network. Adv. Neural Inf. Process. Syst..

[2] Li B., Shen C., Dai Y., Van Den Hengel A., He M. Depth and surface normal estimation from monocular images using regression on deep features and hierarchical crfs. Proceedings of the IEEE Conference on Computer Vision and Pattern Recognition.

[3] Liu F., Shen C., Lin G., Reid I. (2017). Learning depth from single monocular images using deep convolutional neural fields. IEEE Trans. Pattern Anal. Mach. Intell..

[4] Gan Y., Xu X., Sun W., Lin L. Monocular depth estimation with affinity, vertical pooling, and label enhancement. Proceedings of the European Conference on Computer Vision (ECCV).

[5] Xu D., Ricci E., Ouyang W., Wang X., Sebe N. Multiscale continuous crfs as sequential deep networks for monocular depth estimation. Proceedings of the IEEE Conference on Computer Vision and Pattern Recognition.

[6] Godard C., Mac Aodha O., Brostow G.J. Unsupervised monocular depth estimation with left-right consistency. Proceedings of the IEEE Conference on Computer Vision and Pattern Recognition.

[7] Garg R., Bg V.K., Carneiro G., Reid I. Unsupervised CNN for single view depth estimation: Geometry to the rescue. Proceedings of the European Conference on Computer Vision.

[8] Kuznietsov Y., Stuckler J., Leibe B. Semi-supervised deep learning for monocular depth map prediction. Proceedings of the IEEE Conference on Computer Vision and Pattern Recognition.

[9] Cao Y., Wu Z., Shen C. (2018). Estimating depth from monocular images as classification using deep fully convolutional residual networks. IEEE Trans. Circuits Syst. Video Technol..

[10] Cao Y., Wu Z., Shen C. (2020). Monocular depth estimation with augmented ordinal depth relationships. IEEE Trans. Circuits Syst. Video Technol..

[11] Fu H., Gong M., Wang C., Batmanghelich K., Tao D. Deep ordinal regression network for monocular depth estimation. Proceedings of the IEEE Conference on Computer Vision and Pattern Recognition.

[12] Zuo Y., Fang Y., Yang Y., Shang X., Wu Q. (2020). Depth map enhancement by revisiting multi-scale intensity guidance within coarse-to-fine stages. IEEE Trans. Circuits Syst. Video Technol..

[13] Mohaghegh H., Karimi N., Soroushmehr S.R., Samavi S., Najarian K. (2019). Aggregation of rich depth-aware features in a modified stacked generalization model for single image depth estimation. IEEE Trans. Circuits Syst. Video Technol..

[14] Ma J., Zhang H., Yi P., Wang Z. (2020). SCSCN: A separated channel spatial convolution net with attention for single-view reconstruction. IEEE Trans. Ind. Electron..

[15] Farooq Bhat S., Alhashim I., Wonka P. (2020). AdaBins: Depth estimation using adaptive bins. arXiv.

[16] Ranftl R., Bochkovskiy A., Koltun V. (2021). Vision transformers for dense prediction. arXiv.

[17] Shi W., Caballero J., Huszár F., Totz J., Aitken A.P., Bishop R., Rueckert D., Wang Z. Real-time single image and video super-resolution using an efficient sub-pixel convolutional neural network. Proceedings of the IEEE Conference on Computer Vision Pattern Recognition.

[18] Torralba A., Oliva A. (2002). Depth estimation from image structure. IEEE Trans. Pattern Anal. Mach. Intell..

[19] Chun C., Park D., Kim W., Kim C. Floor detection based depth estimation from a single indoor scene. Proceedings of the 2013 IEEE International Conference on Image Processing.

[20] Saxena A., Chung S.H., Ng A.Y. (2005). Learning Depth from Single Monocular Images. Adv. Neural Inf. Process. Syst..

[21] Liu J., Zhang Y., Cui J., Feng Y., Pang L. (2019). Fully convolutional multi-scale dense networks for monocular depth estimation. IET Comput. Vis..

[22] Huang G., Liu Z., Van Der Maaten L., Weinberger K.Q. Densely Connected Convolutional Networks. Proceedings of the 2017 IEEE Conference on Computer Vision and Pattern Recognition (CVPR).

[23] Zioulis N., Alvarez F., Zarpalas D., Daras P. (2022). Monocular spherical depth estimation with explicitly connected weak layout cues. ISPRS J. Photogramm. Remote Sens..

[24] Wofk D., Ma F., Yang T.J., Karaman S., Sze V. Fastdepth: Fast monocular depth estimation on embedded systems. Proceedings of the IEEE International Conference on Robotics and Automation (ICRA).

[25] Sandler M., Howard A., Zhu M., Zhmoginov A., Chen L.C. Mobilenetv2: Inverted residuals and linear bottlenecks. Proceedings of the IEEE Conference on Computer Vision Pattern Recognition.

[26] Russakovsky O., Deng J., Su H., Krause J., Satheesh S., Ma S., Huang Z., Karpathy A., Khosla A., Bernstein M. (2015). Imagenet large scale visual recognition challenge. Int. J. Comput. Vis..

[27] Howard A.G., Zhu M., Chen B., Kalenichenko D., Wang W., Weyand T., Andreetto M., Adam H. (2017). Mobilenets: Efficient convolutional neural networks for mobile vision applications. arXiv.

[28] Zhang X., Zhou X., Lin M., Sun J. ShuffleNet: An extremely efficient convolutional neural network for mobile devices. Proceedings of the 2018 IEEE/CVF Conference on Computer Vision and Pattern Recognition.

[29] Zoph B., Vasudevan V., Shlens J., Le Q.V. Learning transferable architectures for scalable image recognition. Proceedings of the 2018 IEEE/CVF Conference on Computer Vision and Pattern Recognition.

[30] Liu W., Anguelov D., Erhan D., Szegedy C., Reed S., Fu C.Y., Berg A.C. Ssd: Single shot multibox detector. Proceedings of the IEEE European Conference on Computer Vision.

[31] Redmon J., Farhadi A. YOLO9000: Better, Faster, Stronger. Proceedings of the 2017 IEEE Conference on Computer Vision and Pattern Recognition (CVPR).

[32] He K., Zhang X., Ren S., Sun J. Deep Residual Learning for Image Recognition. Proceedings of the 2016 IEEE Conference on Computer Vision and Pattern Recognition (CVPR).

[33] Chen L.C., Papandreou G., Kokkinos I., Murphy K., Yuille A.L. (2018). DeepLab: Semantic image segmentation with deep convolutional nets, Atrous convolution, and fully connected CRFs. IEEE Trans. Pattern Anal. Mach. Intell..

[34] Uhrig J., Schneider N., Schneider L., Franke U., Brox T., Geiger A. Sparsity invariant CNNs. Proceedings of the International Conference on 3D Vision (3DV).

[35] Cordts M., Omran M., Ramos S., Rehfeld T., Enzweiler M., Benenson R., Franke U., Roth S., Schiele B. The cityscapes dataset for semantic urban scene understanding. Proceedings of the IEEE Conference on Computer Vision Pattern Recognition.

[36] Silberman N., Hoiem D., Kohli P., Fergus R. Indoor segmentation and support inference from rgbd images. Proceedings of the European Conference on Computer Vision.

[37] Laina I., Rupprecht C., Belagiannis V., Tombari F., Navab N. Deeper depth prediction with fully convolutional residual networks. Proceedings of the 2016 Fourth International Conference on 3D Vision (3DV).

[38] Wang L., Zhang J., Wang O., Lin Z., Lu H. SDC-Depth: Semantic divide-and-conquer network for monocular depth estimation. Proceedings of the IEEE/CVF Conference on Computer Vision and Pattern Recognition (CVPR).

[39] Hao Z., Li Y., You S., Lu F. Detail preserving depth estimation from a single image using attention guided networks. Proceedings of the International Conference on 3D Vision (3DV).

[40] Lee W., Park N., Woo W. (2011). Depth assisted real-time 3d object detection for augmented reality. ICAT.

[41] Ramamonjisoa M., Lepetit V. Sharpnet: Fast and accurate recovery of occluding contours in monocular depth estimation. Proceedings of the International Conference on Computer Vision.

[42] Hu J., Ozay M., Zhang Y., Okatani T. Revisiting single image depth estimation: Toward higher resolution maps with accurate object boundaries. Proceedings of the 2019 IEEE Winter Conference on Applications of Computer Vision (WACV).

[43] Chen X., Chen X., Zha Z.J. (2019). Structure aware residual pyramid network for monocular depth estimation. arXiv.

[44] Yin W., Liu Y., Shen C., Yan Y. Enforcing geometric constraints of virtual normal for depth prediction. Proceedings of the IEEE/CVF International Conference on Computer Vision.

[45] Lee J.H., Han M.K., Ko D.W., Suh I.H. (2019). From big to small: Multi-scale local planar guidance for monocular depth estimation. arXiv.

[46] Zhang Z., Cui Z., Xu C., Jie Z., Li X., Yang J. Joint task-recursive learning for semantic segmentation and depth estimation. Proceedings of the IEEE European Conference on Computer Vision.

[47] Chollet F. Xception: Deep Learning with depthwise separable convolutions. Proceedings of the 2017 IEEE Conference on Computer Vision and Pattern Recognition (CVPR).

[48] Tan M., Le Q. EfficientNet: Rethinking model scaling for convolutional neural networks. Proceedings of the International Conference on Machine Learning (ICML).

